# HCDFI-YOLOv8: A Transmission Line Ice Cover Detection Model Based on Improved YOLOv8 in Complex Environmental Contexts

**DOI:** 10.3390/s25175421

**Published:** 2025-09-02

**Authors:** Lipeng Kang, Feng Xing, Tao Zhong, Caiyan Qin

**Affiliations:** 1 School of Electrical Engineering, Liaoning University of Technology, Jinzhou 121001, China; lipengkang0512@outlook.com (L.K.);; 2 China Energy Engineering Group Northeast No.3 Electric Power Construction Co., Ltd., Tianjin 300450, China; lfwei2549@ceec.net.cn; 3 School of Mechanical Engineering and Automation, Harbin Institute of Technology, Shenzhen 518055, China

**Keywords:** Transmission line ice-covering detection, YOLO, CDH, C2f_DFF, FASFF, Inner_CIoU

## Abstract

When unmanned aerial vehicles (UAVs) perform transmission line ice cover detection, it is often due to the variable shooting angle and complex background environment, which leads to difficulties such as poor ice-covering recognition accuracy and difficulty in accurately identifying the target. To address these issues, this study proposes an improved icing detection model based on HCDFI–You Only Look Once version 8 (HCDFI-YOLOv8). First, a cross-dense hybrid (CDH) parallel heterogeneous convolutional module is proposed, which can not only improve the detection accuracy of the model, but also effectively alleviate the problem of the surge in the number of floating-point operations during the improvement of the model. Second, deep and shallow feature weighted fusion using improved CSPDarknet53 to 2-Stage FPN_Dynamic Feature Fusion (C2f_DFF) module is proposed to reduce feature loss in neck networks. Third, optimization of the detection head using the feature adaptive spatial feature fusion (FASFF) detection head module is performed to enhance the model’s ability to extract features at different scales. Finally, a new inner-complete intersection over union (Inner_CIoU) loss function is introduced to solve the contradiction of the CIOU loss function used in the original YOLOv8. Experimental results demonstrate that the proposed HCDFI-YOLOv8 model achieves a 2.7% improvement in mAP@0.5 and a 2.5% improvement in mAP@0.5:0.95 compared to standard YOLOv8. Among twelve models for icing detection, the proposed model delivers the highest overall detection accuracy. The accuracy of the HCDFI-YOLOv8 model in detecting complex transmission line environments is verified and effective technical support is provided for transmission line ice cover detection.

## 1. Introduction

As global climate change leads to frequent extreme weather events, the risk of ice on transmission lines may increase, and the importance and urgency of this research will become increasingly prominent [1,2]. Continuously deepening the research on remote sensing technology in accurate icing identification, quantitative inversion, prediction, and early warning are crucial for building a strong, intelligent, green, and safe modern power grid [3,4].

At present, the deep learning object detection algorithms used in the remote sensing field are mainly divided into one-stage and two-stage algorithms [5,6,7]. Among them, the two-stage algorithms mainly include models such as R-CNN, R-FCN, Cascade R-CNN, and DETR. For example, Reference [8] used Faster R-CNN to detect seven dental conditions in DPR images, improving the efficiency and accuracy of dentists’ examinations. Reference [9] pointed out that existing pedestrian detection methods suffer from performance degradation in the presence of small-scale pedestrians who are positioned at a far distance from the camera. It proposed a framework to integrate semantic segmentation into the confidence modules of the RPN (Region Proposal Network) head, R-FCN (Region-based Fully Convolutional Network) head, and cascaded R-FCN head to further improve the performance of the model architecture. Reference [10] proposed a cascaded architecture called 3D Cascade RCNN, which allocates multiple detectors based on voxelized point clouds in a cascaded paradigm to gradually pursue higher-quality 3D object detectors. Reference [11] introduces the Infrared Small Target Detection Transformer (IST-DETR), a new model specifically designed to meet the challenges of low-resolution infrared imagery and small target scales. Excellent results have been achieved in recognizing small infrared targets in different complex scenes. In the Reference [12], for the field of light guide plate (LGP) quality inspection in industrial production, the RT-DETR-LGP model is proposed to adopt the newly designed multi-scale edge information enhancement (MSEIE) module, aggregated diffusion pyramid network (ADPN) module, and the multi-scale feature fusion strategy to achieve efficient detection of defects of different scales. In summary, its two-stage detection model has high accuracy but slow detection speed, which is more suitable for high-precision fields such as medical imaging and industrial quality inspection. In contrast, the one-stage algorithms (TOOD and YOLO series) perform detection in an end-to-end network in one go [13]. Unlike the two-stage algorithms that first propose candidate regions and then classify and refine these candidate regions, the one-stage algorithms greatly reduce the detection time and are more practical for real-time object detection [14].

The YOLO (you only look once) series of models, as outstanding representatives of one-stage detectors, have been continuously innovating and iterating since first deployment and have always been at the forefront of the real-time object detection field [15]. YOLOv8, launched in early 2023 and developed by the Ultralytics team, inherits the efficient genes of the YOLO family [16]. YOLOv8 has improved the accuracy-speed benchmark on general object detection datasets (such as COCO), and has also shown excellent performance potential in the extraction of ground objects from remote sensing images (such as buildings, vehicles, and ships). For example, Reference [17] proposed an indirect-vision-based fire detection method called MITA-YOLO, which integrates indirect vision deployment and an enhanced detection module. It uses mirror angles to achieve indirect views, solves the problem of limited visibility in irregular spaces, and aligns each indirect view with the target monitoring area. Reference [18] proposed a new anchor-free driving scene detection network based onYOLOv8, and the test results show that the performance of the proposed algorithm on the large-scale small object detection dataset (SODA-A) is better than YOLOv8 in terms of speed and accuracy by using DBB module, bidirectional feature pyramid network (BiFPN), and a query-based model with a new pipeline structure. Reference [19] proposed a new component-based detection method for large ship targets in SAR images. After feature extraction using the GELAN network, it integrated multi-level and multi-pool channel attention to optimize the feature extraction structure in a hierarchical manner, effectively improving the detection performance of the model. However, when applying YOLO’s powerful object detection ability to the specific, delicate, and highly valuable engineering task of transmission line icing detection, challenges arise because transmission lines are often distributed in complex mountainous areas and are affected by factors such as illumination, weather, and imaging angles. These challenges are the key points that this research aims to overcome.

In recent years, regarding the research on transmission line icing detection, Reference [20] proposed a YOLOv8-seg algorithm for transmission line icing detection. By introducing deformable convolution v2 (DCNv2) into the Cf2 module at the bottom of the backbone network, introducing the triplet attention mechanism into the network, improving the upsampling method of the network, and applying the sliding weight function to loss calculation, the algorithm was improved. The experimental results showed that the accuracy rates of the improved YOLOv8-seg algorithm were 95.28% and 95.04%, respectively. Reference [21] proposed an icing detection method based on an improved YOLOv8 network to detect and extract the ice-covered areas of ADSS optical cables and reduce the interference of complex backgrounds. Reference [22] designed a feature enhancement module based on atrous convolution to increase the receptive field, obtain more context information while retaining the texture details of the feature map, and improve the accuracy of the proposed algorithm by 7.6% to address the problem that transmission line icing can affect the safety of the power system operation. Reference [23] proposed an improved YOLO8 algorithm to identify the ice-covered image areas of transmission lines. By introducing deformable convolution DCNv3 to generate learnable offsets and improving the traditional fixed convolution operation, it realized non-linear sampling of the input feature map during the convolution process, improving the robustness and accuracy of the algorithm. Then, it introduced the SIoU loss function to optimize the network, and the experimental results achieved an inference accuracy of 88.8% mAP.

In summary, although the above articles have achieved certain improvements in the accuracy of transmission line icing detection, there are problems such as an increase rapidly in the number of floating-point operations during model improvement, feature loss when fusing local feature information, weak ability to extract features of different scales, and limitations of the original loss function. To solve the above problems, this paper proposes an HCDFI-YOLOv8 model structure to provide technical reference for transmission line icing detection using remote sensing.

The main innovations and contributions of this paper are as follows: (1) A CDH (cross-dense hybrid) module is proposed to improve the backbone network. By designing a parallel heterogeneous kernel convolution to extract feature information, the detection accuracy of the model can be improved and the problem of the surge in the number of floating-point operations can be effectively mitigated during the model improvement process. (2) In the neck network, using the improved C2f_DFF (CSPDarknet53 to 2-Stage FPN_Dynamic feature fusion) module in this paper, the shallow and deep features in the network are weighted and fused to reduce the neck network feature loss. (3) A head network composed of FASFF (feature adaptive spatial feature fusion) modules is built. By an adaptive spatial feature fusion strategy, the ability of the head network to extract features of different scales is enhanced. (4) A new Inner_CIoU (inner-complete intersection over union) loss function is adopted. Through the auxiliary box scale dynamic scaling mechanism, the limitations of the YOLOv8 model’s own loss function are solved.

The chapter arrangement is as follows. In Section 2, aiming at the problems of false detection and low accuracy during transmission line icing detection, which are easily affected by multiple factors such as varying angles and complex environmental backgrounds, an HCDFI-YOLOv8 transmission line icing detection model is proposed, and its improved modules are presented. In Section 3, experimental results and analyses were carried out. The validity of the improved model was verified by comparing the ablation experimental data with the visualized images. The superiority of the model proposed in this paper is further verified through the comparative experimental analysis of each model. Finally, in Section 4, there is a summary of conclusions, a presentation of the shortcomings of this paper, and a look ahead.

## 2. Transmission Line Icing Detection Model Based on HCDFI-YOLOv8

**Figure 1:**
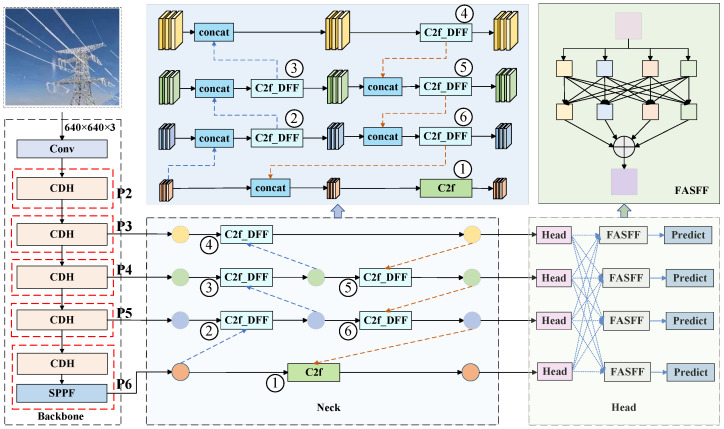
Architecture of the HCDFI-YOLOv8 model.

When performing the transmission line icing detection task using YOLOv8, the detection process is often affected by factors such as varying angles and complex environmental backgrounds, resulting in problems such as false detections and low accuracy during icing detection. Therefore, this paper proposes an HCDFI-YOLOv8 model structure, which improves the backbone network, neck network, head network, and loss function of YOLOv8, respectively. The model architecture is shown in Figure 1.

In the backbone network, P2, P3, P4, P5, and P6 are composed of the CDH module proposed in this paper, which adopts a small convolutional kernel instead of part of the large convolutional kernel to effectively alleviate the problem of the proliferation of the number of floating-point operations in the process of improving the model and to improve the detection accuracy of the model. Additionally, this is performed because of the use of the four detection heads in this paper, and so compared to the model with the YOLOv8 one more P6 layer is added. In the neck network, the improved C2f_DFF module is used to replace the C2f module, which can perform dynamic feature adjustment and helps improve the detection accuracy of the model. In the head network, the improved four detection head FASSF module is used. The weights of four levels are obtained through adaptive network learning, and finally, the features are normalized to generate the final features, achieving the purpose of multi-level feature fusion. Finally, due to the limitations of the original loss function, the loss function Inner_CIoU is introduced. It calculates the loss of the model using auxiliary boxes of different scales, enabling the model to converge faster and more effectively and further improving the accuracy of the model in detecting icing on transmission lines.

### 2.1. CDH Optimization Module Based on the Dual Idea

**Figure 2:**
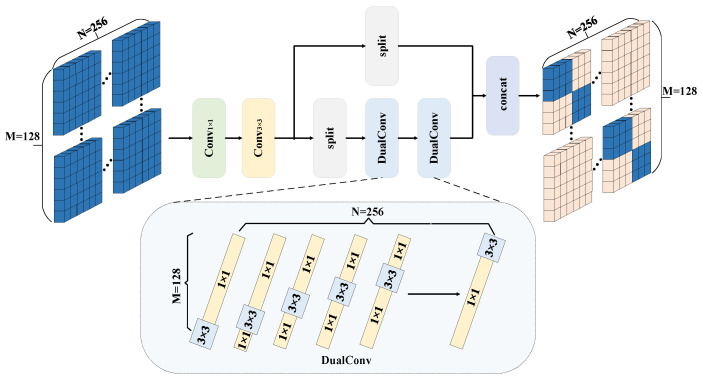
CDH module architecture.

This paper addresses the issue that the YOLOv8 model is prone to a surge in the number of floating-point operations of the model while improving the accuracy of the model detection. To do so, a CDH module is proposed to be applied in the backbone network, and its architecture is shown in Figure 2. Compared with the traditional standard convolutional kernel module, this module uses the dual idea and adopts the characteristics of parallel heterogeneity for the kernel convolutional module, improving the performance of YOLOv8.

**Figure 3:**
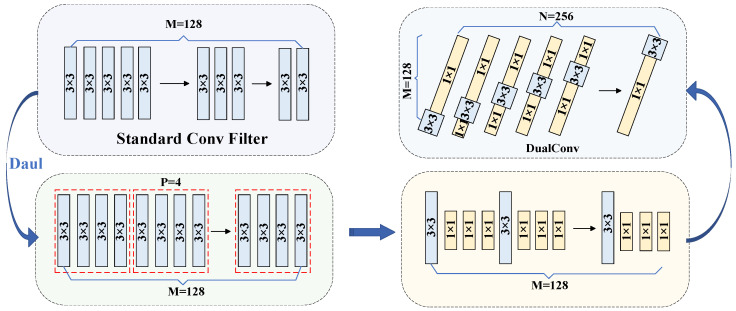
Transformed DualConv module.

M represents the number of channels of the input feature map, and N represents the number of channels of the output feature map. It internally contains a convolution module, a tensor splitting module, a DualConv module, and a tensor splicing module. It uses parallel heterogeneous convolution groups to capture rich contextual information, effectively reducing the computational load and model complexity while maintaining the quality of feature fusion. The transformation process of its DualConv module is shown in Figure 3.

In Figure 3, P represents the number of convolutional kernels divided into parts, and dual refers to the use of two convolutional kernel (1 × 1 and 3 × 3) paths to process the information in order to improve the detection performance of the model. First, the DualConv module is based on the YOLOv8 standard conv filter. Using the idea of dual, the convolution kernels of 128 input channels are divided into four parts. Secondly, one part of the four parts is a 3 × 3 convolution kernel, and the other three parts are composed of 1 × 1 convolution kernels in parallel. By replacing 3/4 of the 3 × 3 convolution kernels with smaller 1 × 1 convolution kernels, the number of floating-point operations can be reduced, thereby accelerating the calculation efficiency of the model. Finally, each output channel adopts this parallel heterogeneous convolution kernel method, and it is applied to the P2, P3, P4, P5, and P6 structures of the backbone network.

The CDH optimization module used in this paper not only alleviates the surge in the number of floating-point operations during the improvement of the model, but also improves the detection accuracy of the model, with the number of floating-point operations calculated as follows:

(1) $$FLOPs=H_{out}\times W_{out}\times C_{out}\times K_{h}\times K_{w}\times C_{in}$$

where H*_out_* and W*_out_* represent the height and width of the output feature map, C*_out_* and C*_in_* represent the number of channels of the output and input feature maps, and K*_h_* and K*_w_* represent the height and width of the convolution kernel, respectively. In this paper, the optimized CDH module, H*_out_*, Wout, and Cout are always the same during the optimization process due to the use of a 1 × 1 convolutional kernel to replace the original 3/4 of the 3 × 3 convolutional kernel; therefore, there will be 3/4 C*_in_* of K*_h_* × K*_w_* from the original 3 × 3 to 1 × 1. In summary, the use of the optimized CDH module in the backbone network is able to effectively alleviate the model’s problem of the surge in the number of floating-point operations during the improvement process.

### 2.2. Improved C2f Feature Extraction Module Based on DFF Attention Guidance

**Figure 4:**
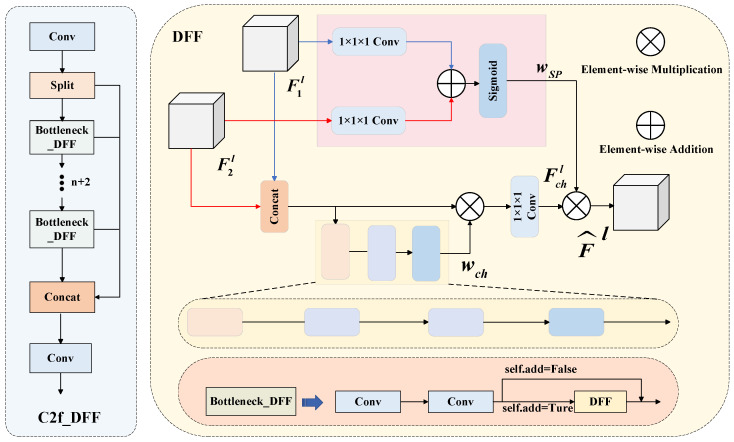
Architecture of C2f_DFF module.

Regarding the problem that local feature information is easily lost when the YOLOv8 model fuses feature information, this paper uses an improved C2f_DFF module in the neck network, as shown in Figure 4. Compared with the C2f module, this module can further enhance the effective utilization of global feature information and improve the detection accuracy of the model for icing on transmission lines on the premise of better retaining local feature information through dynamic fusion.

**Figure 5:**
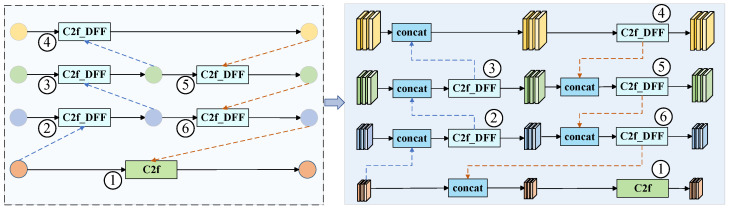
Improved neck network architecture.

In Figure 4, B represents the number of batch sizes (B = 20), C represents the number of channel dimensions (C = 256), 1 × 1 × 1 convolution represents the standard 1 × 1 convolution, and the number of filters is 1. The ***F_1_^l^*** of the DFF module is the input directly from the current bottleneck block through split, and the ***F_2_^l^*** is the feature from inside the current bottleneck block processed by two 1 × 1 × 1 convolutions as input. The DFF block works as follows: firstly, a branch of both ***F_1_^l^*** and ***F_2_^l^*** input feature maps are 1 × 1 × 1 convolved for spatial dimensional feature refinement, and the two are summed up to generate the spatial weight wsp through the activation of the Sigmoid function to emphasize the important spatial regions. Secondly, another branch of the ***F_1_^l^*** and ***F_2_^l^*** input feature maps is spliced by Concat, and the multi-scale pooling operation is performed through AVGPool to output a one-dimensional vector of [20, 512, 1, 1], which successively undergoes two 1 × 1 convolutions for the process of dimensionality reduction and reshaping. This keeps the computational map of the whole network mainly composed of convolutional operations, which simplifies the implementation and optimization. The Sigmoid function activation generates the channel weight ***w_ch_***, which is combined with the input features by element-wise multiplication, followed by one 1 × 1 × 1 convolution to obtain the channel weight ***F_ch_^l^***. Finally, the spatial weight ***w_sp_*** and the channel weight ***F_ch_^l^*** are combined through element-wise multiplication to achieve residual connection, and the enhanced features are finally output. The C2f_DFF module used in this paper is an improvement based on C2f. By using the DFF module [24,25,26], multi-scale information can be combined more effectively, and the loss of local feature information during model fusion can be reduced, thereby improving the detection accuracy of the model for ice-covered transmission lines. In this paper, the improved C2f_DFF module is applied to the neck network, and its improved neck network structure is shown in Figure 5.

In Figure 5, ①, ②, ③, ④, ⑤, and ⑥ represent the position of the original neck network C2f, the blue dotted line represents the upsample, and the yellow curve represents the Conv module. The left diagram in Figure 5 is a simplified diagram of the improved neck network structure, and the right diagram is the specific structure diagram of the improved neck network, and the two are equivalent. The improved neck network in this paper uses multi-level interlayer interaction to diffuse features with rich contextual information, which enables the neck network to perform more flexible feature fusion between features of different scales, effectively improves the overall feature fusion ability of the model, and is more conducive to the subsequent ice detection and classification tasks of transmission lines.

### 2.3. FASFF Detection Head Module Based on Dynamic Fusion of Multi-Scale Features

**Figure 6:**
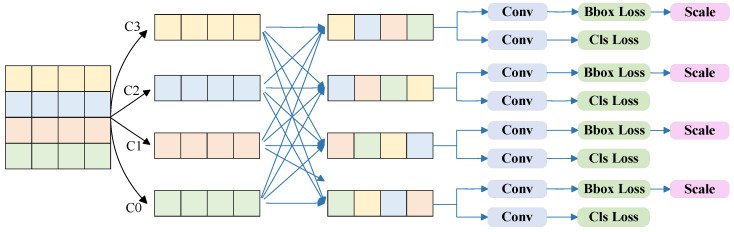
FASFF detection head architecture.

When detecting icing on transmission lines, there are differences in size due to the distance difference, but the characteristics of icing have scale invariance. Therefore, this paper adopts an improved adaptive spatial feature fusion strategy (FASFF) [27,28,29] to solve the inconsistency problem between different feature scales. In order to make more full use of the characteristics of icing and improve the accuracy of ice-coating detection on transmission lines, this paper replaces the detection head with a four-head FASFF detection head, and its principle is shown in Figure 6.

In Figure 6, C0, C1, C2, and C3 represent the largest, larger, medium, and smallest detection layers, respectively. For the feature representation of a specific layer, first, cross-layer feature aggregation and scale alignment operations are required (unifying the features of different layers to the same resolution), and then a learnable adaptive spatial weight matrix (optimized through end-to-end training) is used to dynamically fuse multi-scale features. In this process if there are semantic conflicts in the features at the spatial position their weights will be suppressed, while the more discriminative features (such as the complementary combination of high-level semantic information and low-level detail features) will dominate through weight enhancement, ultimately achieving collaborative optimization between features and noise filtering.

The feature fusion of the FASFF detection head for the l-th layer is as follows [30]:

(2) $$y_{ij}^{l}=\alpha_{ij}^{l}\cdot x_{ij}^{1\to l}+\beta_{ij}^{l}\cdot x_{ij}^{2\to l}+\gamma_{ij}^{l}\cdot x_{ij}^{3\to l}+\delta_{ij}^{l}\cdot x_{ij}^{4\to l}$$

where $x_{ij}^{n\to l}$ (n = 1, 2, 3 and 4) represents the features of the nth layer after resolution alignment to the target level l; *α^l^_ij_*, *β^l^_ij_*, *γ^l^_ij_*, and *δ^l^_ij_* refer to the four weights obtained through the learning of the adaptive neural network, which are optimized and learned together with other network parameters during training through backpropagation and gradient descent; and *y^l^_ij_* represents the feature value at position l in the output feature map after fusion. This multi-feature fusion is suitable for tasks that require the combination of multi-scale information. FASFF fuses features from four different levels to the same resolution through adaptive weights and generates the final features through normalization or residual processing, achieving fine-grained fusion of multi-level features and further improving the detection accuracy of the model for icing on transmission lines in complex scenarios.

### 2.4. Improvement of Inner-CIoU Loss Based on Inscribed Rectangle

The loss function can accelerate the convergence speed of the model. YOLOv8 uses the CIoU loss function [31,32], which has limitations due to the neglect of the IoU loss term itself. Therefore, this paper uses an improved Inner_CIoU [33,34] loss function. This loss function can not only solve its own limitations but also distinguish different regression samples. It calculates the loss of the model using auxiliary boxes of different scales, thus accelerating the convergence speed of the model more quickly and effectively.

As an important part of the existing mainstream bounding box regression loss functions, IoU is defined as follows [35]:

(3) $$IoU=\frac{\left|B\cap B^{p}\right|}{\left|B\cup B^{p}\right|}$$

where *B* represents the ground truth box, *B^P^* represents the prediction box, and the corresponding loss can be expressed as [36]:

(4) $$L_{IoU}=1-IoU$$

**Figure 7:**
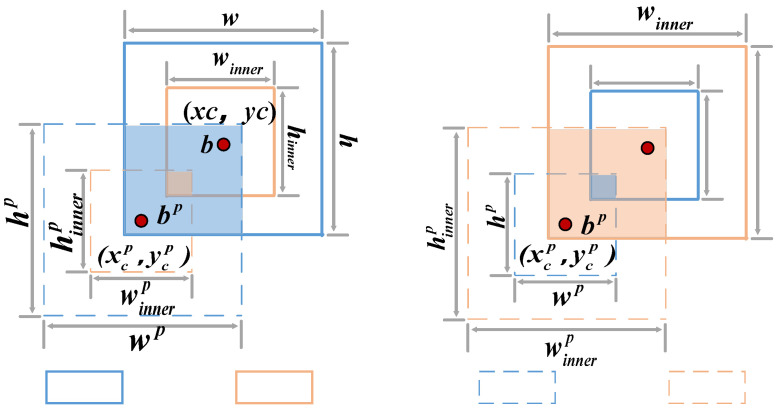
Description of inner-IoU.

To address the issue of the slow convergence speed of the model in the detection of ice-covered transmission lines in different environments using the IoU loss function, this paper introduces inner-IoU. By using auxiliary bounding boxes of different scales for different datasets and detectors, as shown in Figure 7, the limitations of the IoU loss function can be effectively overcome.

In Figure 7, the solid-line box represents the ground truth box; the dashed-line box represents the anchor box; (*x_c_*,*y_c_*) represents the center point of the anchor box and the center point of the inner anchor box; (*x_c_^p^*,*y_c_^p^*) represents the center point of the real frame and the center point of the inner real frame; *w* and *h* represent the width and height of the real box, respectively; *w^p^* and *h^p^* represent the width and height of the anchor box, respectively.; *w_inner_* and *h_inner_* represent the width and height of the inner real box, respectively; and *w^p^_inner_* and *h^p^_inner_* represent the width and height of the inner anchor box, respectively. The calculation method of the Inner_CIoU loss function is as follows [37,38]:

(5) $$b_{l}=x_{c}-\frac{w\times ratio}{2},b_{r}=x_{c}+\frac{w\times ratio}{2}$$

(6) $$b_{t}=y_{c}-\frac{h\times ratio}{2},b_{b}=y_{c}+\frac{h\times ratio}{2}$$

(7) $$b_{l}^{p}=x_{c}^{p}-\frac{w^{p}\times ratio}{2},b_{r}^{p}=x_{c}^{p}+\frac{w^{p}\times ratio}{2}$$

(8) $$b_{t}^{p}=y_{c}^{p}-\frac{h^{p}\times ratio}{2},b_{b}^{p}=y_{c}^{p}+\frac{h^{p}\times ratio}{2}$$

(9) $$inter=(min(b_{r},b_{r}^{p})-max(b_{l},b_{l}^{p}))\times (min(b_{b},b_{b}^{p})-max(b_{t},b_{t}^{p}))$$

(10) $$union=(w\times h)\times {\left(ratio\right)}^{2}+(w^{p}\times h^{p})\times {\left(ratio\right)}^{2}-inter$$

(11) $$IoU^{inner}=\frac{inter}{union}$$

Then the Inner_CIoU is:

(12) $$L_{Inner\_CIoU}=1+\frac{\rho^{2}(b,b^{p})}{c^{2}}-IoU^{inner}+\alpha v$$

where ρ represents the distance between points b and b^p^; c represents the diagonal length of the smallest bounding box that encloses both the predicted bounding box and the ground truth box; α represents a positive trade-off parameter; and *v* measures the consistency of the aspect ratio.

## 3. Experimental Results and Analysis

### 3.1. Datasets and Experimental Environment

**Figure 8:**
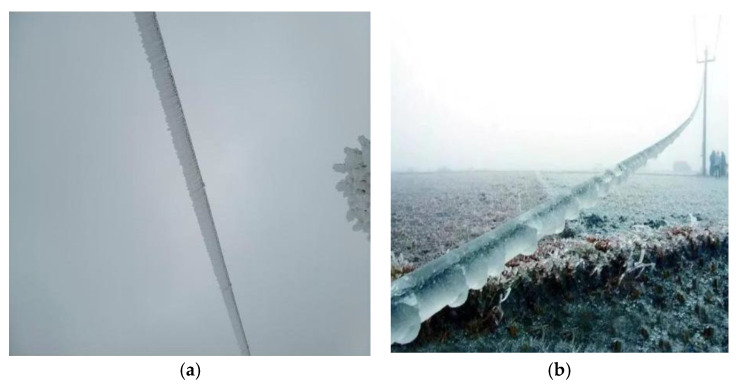
Dataset images. (**a**) Mixed rime; (**b**) glaze.

The transmission line icing detection dataset selected in this study is from the public dataset on CSDN [39]. The dataset contains a total of 1372 images, including 1144 images in the training set and 228 images in the validation set. There are two categories, mixed rime (opaque mixed crystals of ice and snow) and glaze (transparent ice), as shown in Figure 8.

The dataset for model training contains icing images of transmission lines with different sizes, types, and background environments. The dataset has the characteristic of diversity, which can enhance the robustness of the model and make it highly practical in multiple scenarios [40].

In this study, the same computer configuration was used for each experiment, and the specific parameters are shown in Table 1.

During training, the input image size is 640 × 640, the batch size is 20, and the epoch is 300. The HCDFI-YOLOv8 in this paper follows the hyperparameters established by the YOLOv8 model. The main hyperparameters and their values are shown in Table 2.

### 3.2. Evaluation Indicators

In this study, five indicators are mainly used to evaluate the network performance, including precision (P), recall rate (R), mean average precision at 50 (mAP@0.5), mean average precision from 50 to 95 (mAP@0.5:0.95), FLOPs, and inference time. The formulas for the P, R, mAP@0.5, and mAP@0.5:0.95 indicators are as follows [41,42]:

(13) $$P=\frac{TP}{TP+FP}$$

(14) $$R=\frac{TP}{TP+FN}$$

where in Formula (13) TP represents the number of positive samples that are correctly predicted and FP represents the number of positive samples that are incorrectly predicted. In Formula (14), FN represents the number of positive samples that are actually positive but not detected.

(15) $$AP=\int_{0}^{1}P(R)dR$$

(16) $$mAP@0.5=\frac{1}{N}\sum_{k=1}^{N}AP@0.5_{k}$$

(17) $$mAP@0.5:0.95=\frac{1}{10}\sum_{k=0}^{9}mAP@0.5+0.05k$$

In Formulas (15)–(17), AP represents the average precision, which is used to evaluate the detection effect of a single category. mAP@0.5 represents the average precision of all categories when the IoU threshold is 0.5, which reflects the ability of the model to maintain high precision at high recall rates. mAP@0.5:0.95 represents the average mAP at different IoU thresholds (from 0.5 to 0.95, with a step size of 0.05), which can more comprehensively reflect the performance of the model under different IoU standards.

R, mAP@0.5, and mAP@0.5:0.95 are used to measure the detection accuracy of the model, and the larger their values, the better. FLOPs is an important indicator for measuring the computational complexity of the model, representing the number of floating-point operations that the model needs to perform during the inference process. Inference time is the time it takes for a trained model to make a prediction and come up with a result on a new, never-before-seen piece of data (an image).

### 3.3. Experimental Comparative Analysis

#### 3.3.1. Analysis of Hyperparameter Selection

**Figure 9:**
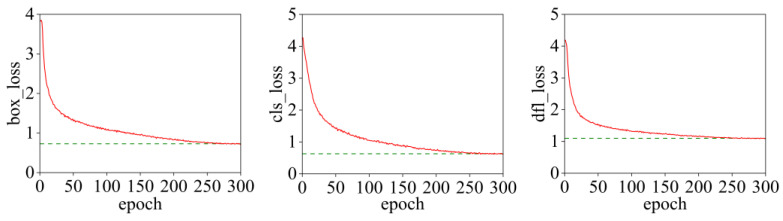
Training loss decreasing process.

**Figure 10:**
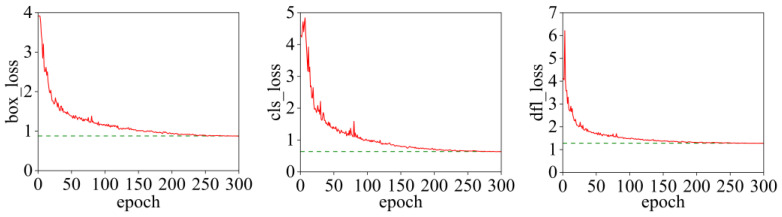
Verification of loss reduction process.

Hyperparameters are crucial for improving the performance of the model. In this paper, the number of epochs is set to 300. The declines in training loss and validation loss are shown in Figure 9 and Figure 10, respectively.

In Figure 9 and Figure 10, the model loss decreases rapidly in the first 50 rounds; between the 250th and 300th rounds, the model loss tends to converge stably without overfitting or underfitting. Therefore, the number of epochs is selected as 300.

#### 3.3.2. Model Performance Analysis

**Figure 11:**
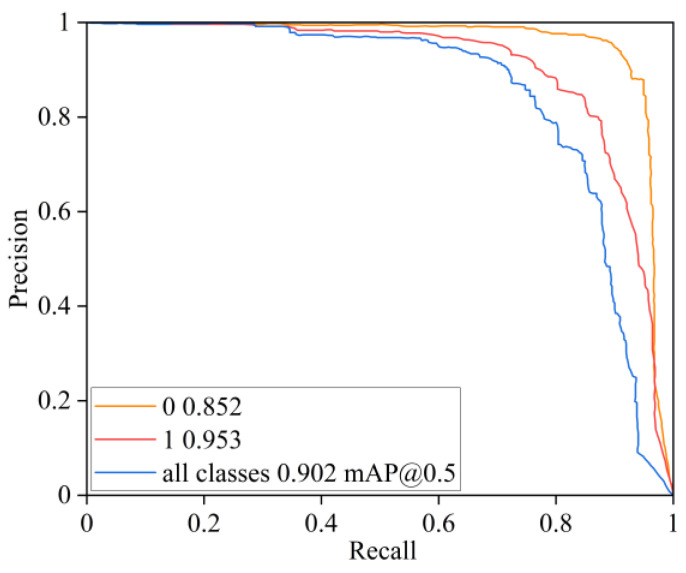
Precision–recall curve diagram.

Model performance is a criterion for measuring the quality of a model. Especially when dealing with imbalanced datasets, the precision–recall curve is commonly used to evaluate the performance of classification models. In this paper, the precision–recall curve of the HCDF-YOLOv8 model is shown in Figure 11. The light blue curve represents the precision–recall curve of mixed rime (“0”); the orange curve represents the precision–recall curve of glaze (“1”); and the dark blue curve represents the precision–recall curve of the overall average (“All”). As recall increases, precision gradually decreases, and the mean average precision (mAP@0.5) of all categories is 0.902, indicating that the overall performance of the model is good.

#### 3.3.3. Analysis of the Position Comparison of C2f_DFF Addition

In order to verify that the improved C2f_DFF used at six locations in Figure 1 has better effects compared with other addition methods, eight groups of experimental comparisons, as shown in Table 3, were carried out.

It can be concluded from Table 3 that in this paper adding the C2f_DFF module at positions ②, ③, ④, ⑤, and ⑥ has the highest accuracy compared with other addition methods.

### 3.4. Ablation Experiment Analysis

To verify the effectiveness of the improved method in this paper, based on the YOLOv8 algorithm, mAP@0.5, mAP@0.5:0.95, and FLOPs were used as evaluation indicators. Ablation experiments were conducted on the dataset using CDH(H), C2f_DFF(CD), FASFF(F), and Inner_CIoU(I) to verify the improvement effect of each module on the model’s detection ability. The experimental results are shown in Table 4.

**Figure 12:**
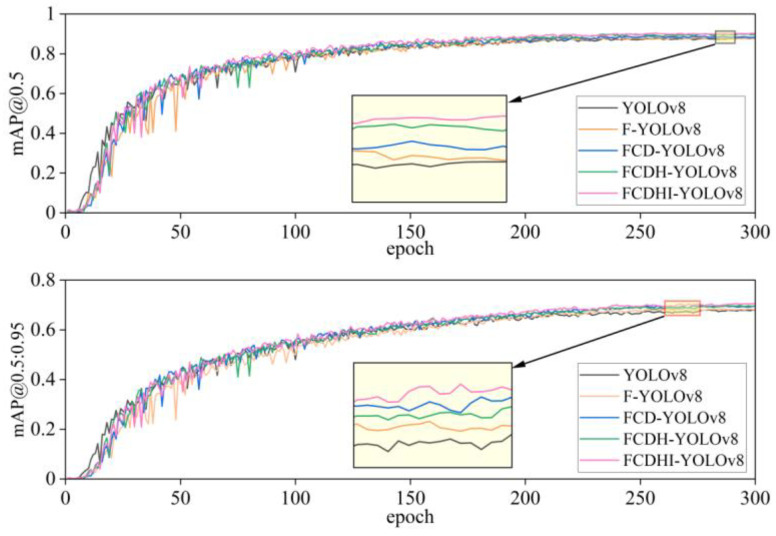
Comparison of mAP@0.5 and mAP@0.5:0.95.

**Figure 13:**
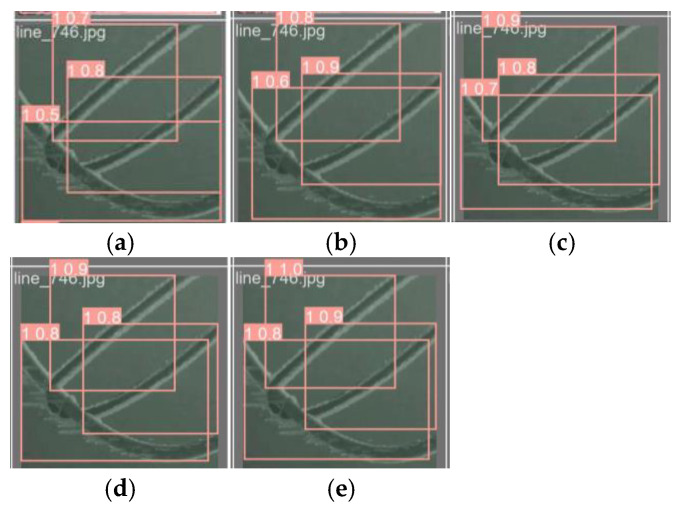
Comparative visualization of the modules. (**a**) YOLOv8; (**b**) F-YOLOv8; (**c**) FCD-YOLOv8; (**d**) FCDH-YOLOv8; (**e**) FCDHI-YOLOv8.

From Table 4, it can be concluded that the YOLOv8 model is 87.5%, 81.8%, and 93.2% on “All”, “0”, and “1” at mAP@0.5, and 68.1%, 62.8%, and 73.4% on “All”, “0”, and “1” at mAP@0.5:0.95, respectively, and the complexity FLOPs of the model are 8.2G. When the FASFF module was introduced alone, FLOPs only improved by 2.1G, increased by 2.0% on “0” of mAP@0.5, and for “1” it decreased by 1.1%, and it increased by 1.5% on “0” of mAP@0.5:0.95, while for “1” it decreased by 0.7%, but for “All” it increased by 0.5% and 0.4% on mAP@0.5 and mAP@0.5:0.95, respectively. When introducing C2f_DFF modules alone, it remains unchanged on “All” at mAP@0.5 and improves by 0.5% on “All” at mAP@0.5:0.95, while FLOPs remain unchanged. When combined with the FASFF module, it improves by 1.1%, 2.0%, and 0.2% on “All”, “0”, and “1” at mAP@0.5, and 1.7%, 1.8%, and 1.5% on “All”, “0”, and “1” at mAP@0.5:0.95. When the CDH module is introduced alone, the “All” of mAP@0.5 increases by 0.5%, the “All” of mAP@0.5:0.95 remains unchanged, and the FLOPs decreases by 0.9G. When combined with FASFF and C2f_DFF modules, it increases by 2.6%, 3.7%, and 1.5% on “All”, “0”, and “1” of mAP@0.5, respectively, and “All”, “0”, and “1” at mAP@0.5:0.95 increased by 1.8%, 1.8%, and 1.7%, respectively. When the Inner_CIoU loss function was introduced alone, the “All” of mAP@0.5 and mAP@0.5:0.95 increased by 0.5% and 0.4%, respectively, and the FLOPs remained unchanged. When combined with FASFF, C2f_DFF, and CDH modules, it improves by 2.7%, 3.4%, and 2.1% on “All”, “0”, and “1” at mAP@0.5, and 2.5%, 2.5%, and 2.5% on “All”, “0”, and “1” at mAP@0.5:0.95, respectively. These results suggest that the proposed modules are not only effective when used alone but also show synergy when combined. The synergistic effects of each module are illustrated in Figure 12 and Figure 13.

In Figure 12, the black curve represents YOLOv8; the yellow curve represents F-YOLOv8; the blue curve represents FCD-YOLOv8; the green curve represents FCDH-YOLOv8; and the pink curve represents the FCDHI-YOLOv8 model proposed in this paper. From the figure, it can be more intuitively seen that the mAP@0.5 and mAP@0.5:0.95 curve trends of the FCDHI-YOLOv8 model proposed in this paper are the best compared with the YOLOv8, F-YOLOv8, FCD-YOLOv8, and FCDH-YOLOv8 models.

In Figure 13, each detection box has two parts: the first part is the detection class and the second part is the detection accuracy. The effectiveness of the FASSF, C2f_DFF, CDH, and Inner_CIoU modules used in this paper are more clearly seen in the five visualizations.

### 3.5. Analysis of Comparative Experiments

For further verification, the improved model is compared with deep learning models commonly used for transmission line ice-cover detection for the P, R, mAP@0.5, FLOPs, and inference time of the models, as shown in Table 5.

In Table 5, compared with the TOOD model, the FCDHI-YOLOv8 model proposed in this paper has 1.5%, 6.2%, and 4.2% higher accuracy in P, R, and mAP@0.5, respectively, and the FLOPs are reduced by 113.6G. Compared with the Faster R-CNN model, it has 2.3%, 5.6%, and 3.9% higher accuracy in P, R, and mAP@0.5, respectively, and the FLOPs are reduced by 124.6G. Compared with the RTDETR-L model, it has 3.7%, 6.4%, and 5.8% higher accuracy in P, R, and mAP@0.5, respectively, and the FLOPs are reduced by 22.6G. Compared with the RTDETR-R18 model, it has 3.5%, 6.0%, and 7.2% higher accuracy in P, R, and mAP@0.5, respectively, and the FLOPs are reduced by 10.5G. Compared with the YOLOv3-tiny model, it has 7.9%, 8.5%, and 10.4% higher accuracy in P, R, and mAP@0.5, respectively, and the FLOPs are reduced by 9.6G. Compared with the YOLOv6 model, it has 5.0%, 11.1%, and 9.3% higher accuracy in P, R, and mAP@0.5, respectively, and the FLOPs are reduced by 2.5G.

Although the FLOPs of the FCDHI-YOLOv8 model proposed in this paper are only 2.2G, 2.1G, 1.2G, 1.2G, and 3.1G higher than those of the YOLOv5, YOLOv5-P6, YOLOv8, YOLOv10, and YOLOv11 models, respectively, compared with the YOLOv5 model it has 5.7%, 2.1%, and 4.3% higher accuracy in P, R, and mAP@0.5, respectively. Compared with the YOLOv5-P6 model, it has 3.1%, 9.5%, and 6.6% higher accuracy in P, R, and mAP@0.5, respectively. Compared with the YOLOv8 model, it has 1.0%, 2.9%, and 2.7% higher accuracy in P, R, and mAP@0.5, respectively. Compared with the YOLOv10 model, it has 4.9%, 7.4%, and 5.1% higher accuracy in P, R, and mAP@0.5, respectively. Compared with the YOLOv11 model, it has 1.6%, 5.1%, and 3.5% higher accuracy in P, R, and mAP@0.5, respectively. For the inference time metric, the smaller its value, the better. Although the inference time of FCDHI-YOLOv8 proposed in this paper is not the fastest, it is similar. Overall, the overall performance of FCDHI-YOLOv8 is the best.

**Figure 14:**
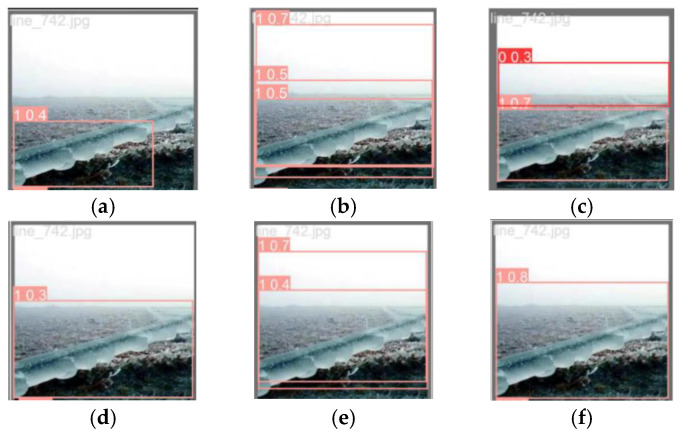
Visual comparison chart of different algorithms for glaze case. (**a**) YOLOv3-tiny; (**b**) YOLOv5; (**c**) YOLOv5-P6; (**d**) YOLOv6; (**e**) YOLOv8; (**f**) FCDHI-YOLOv8.

**Figure 15:**
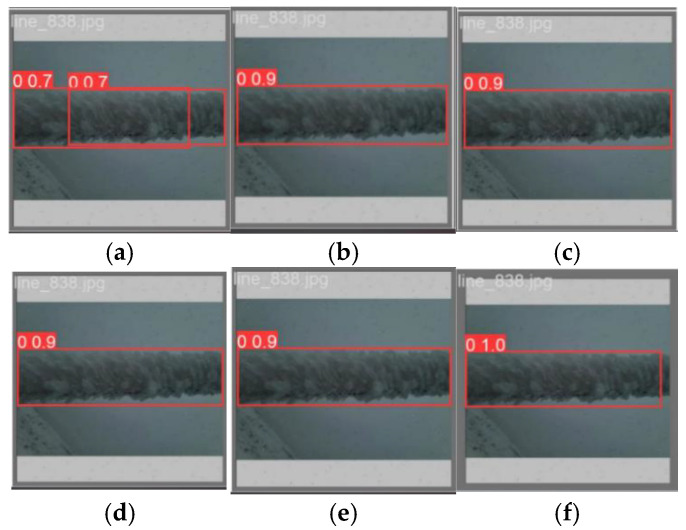
Visual comparison chart of different algorithms for mixed rime case. (**a**) YOLOv3-tiny; (**b**) YOLOv5; (**c**) YOLOv5-P6; (**d**) YOLOv6; (**e**) YOLOv8; (**f**) FCDHI-YOLOv8.

In the comparative experiment, five models, namely YOLOv3-tiny, YOLOv5, YOLOv5-P6, YOLOv6, and YOLOv8, were selected to be compared with the visualized images of the FCDHI-YOLOv8 model proposed in this paper, as shown in Figure 14 and Figure 15.

It can be seen from Figure 14 that the detection accuracies of the five selected comparison models are all lower than the accuracy of the FCDHI-YOLOv8 model proposed in this paper, and that there are false detection phenomena in YOLOv5, YOLOv5-P6, and YOLOv8. It can be seen from Figure 15 that there are false detections and low detection accuracy in YOLOv3-tiny, and the detection accuracies of the other four models are all lower than that of the FCDHI-YOLOv8 model. Therefore, it further shows that the FCDHI-YOLOv8 model proposed in this paper can effectively reduce the problems of false detection and low detection accuracy of models in complex environmental backgrounds.

In general, the FCDHI-YOLOv8 model proposed in this paper can effectively improve the accuracy of transmission line ice-coating detection.

## 4. Conclusions

This paper addresses the problems that often occur during the icing detection task of power transmission lines using YOLOv8. In the detection process, it is frequently affected by factors such as varying angles and complex environmental backgrounds, leading to issues like false detections and low accuracy in icing detection. A power transmission line icing detection model named FCDHI-YOLOv8 is proposed.

Firstly, a CDH module is proposed in the backbone network, which can not only effectively mitigate the surge in the number of floating-point operations in the model during the improvement process but also enhance the detection accuracy of the model. Secondly, the DFF module is used to improve the C2f in the neck network, reducing the problem of easy information loss during local feature fusion in the model. In addition, a four-detection-head FASFF is adopted in the head network. By fusing features at different levels, the model’s detection ability for targets of different scales is enhanced. Finally, the Inner_CIoU loss based on the inscribed rectangle is introduced to overcome its own limitations.

The experimental results show that compared with the baseline algorithm YOLOv8, the FCDHI-YOLOv8 model proposed in this paper improves by 2.7% and 2.5% in mAP@0.5 and mAP@0.5:0.95, respectively. Compared with the common power transmission line icing detection models, such as TOOD, Faster R-CNN, RTDETR-L, RTDETR-R18, YOLOv3-tiny, YOLOv5, YOLOv5-P6, YOLOv6, YOLOv10, and YOLOv11, the mAP@0.5 is increased by 4.2%, 3.9%, 5.8%, 7.2%, 10.4%, 4.3%, 6.6%, 9.3%, 5.1%, and 3.5%, respectively. Further verification shows that the FCDHI-YOLOv8 model proposed in this paper has good research significance for the real-time detection of icing on power transmission lines. Despite the significant improvements made by FCDHI-YOLOv8, there are still some limitations. This paper uses a limited number of datasets with relatively simple backgrounds. For models under different weather conditions or in different terrains, the performance may be adversely affected. In addition, the aspect ratios of transmission line objects are relatively large, and the model’s dependence on high-quality labeled data during training is critical.

To address these challenges, the outlook for the future is twofold. Firstly, to build a fusion network of “image + meteorological time series data + 3D terrain”. Spatial and environmental factors are associated through neural networks to enhance the robustness of the model in the face of different weather and complex environments. Secondly, OBB will be used to produce a more accurate ice cover dataset to further improve the accuracy of the model in detecting transmission line ice cover.

## Figures and Tables

**Table 1:** Training platform information.

Computer Configuration	Parameter Information
CPU	Intel Core i7 14650HX
GPU	NVIDIA GeForce RTX 4060 Laptop
Operating system	Windows11
Python version	3.9.21
Pytorch version	2.1.0

**Table 2:** HCDFI-YOLOv8 main hyperparameter values.

Hyperparameterisation	Parameter Information
Initial learning rate (lr0)	0.01
Final OneCycleLR learning rate (lrf)	0.01
SGD optimizer momentum	0.937
Optimizer weight-decay	0.0005
Warmup epochs	3.0
Warmup initial momentum	0.8
Warmup initial bias lr	0.1
Box loss gain	7.5
Cls loss gain	0.5
Obj loss gain	1.0

**Table 3:** Experimental comparison of adding C2f_DFF at different positions.

Experiment	C2f_DFF Addition Position						mAP@50/(%)	mAP@50:95/(%)	FLOPs/(G)
	①	②	③	④	⑤	⑥			
1	√	√	√	√	√	√	89.2	68.3	9.4
2	-	-	√	√	√	√	89.3	68.6	9.4
3	-	-	-	√	√	√	87.7	67.7	9.4
4	-	-	-	-	√	√	89.8	70.2	9.4
5	-	-	√	-	√	-	89.4	69.4	9.4
6	-	√	-	-	-	√	89.3	68.2	9.4
7	-	-	-	-	-	√	88.5	68.4	9.4
8	-	√	√	√	√	√	90.2	70.6	9.4

**Table 4:** Comparison table of ablation experiment data.

YOLOv8	F	CD	H	I	mAP@0.5/(%)			mAP@0.5:0.95/(%)			FLOPs/(G)
					All	0	1	All	0	1	
√					87.5	81.8	93.2	68.1	62.8	73.4	8.2
√	√				88.0	83.8	92.1	68.5	64.3	72.7	10.3
√		√			87.5	82.2	92.8	68.6	64.1	73.1	8.2
√			√		88.0	82.3	93.8	68.1	63.3	72.9	7.3
√				√	88.0	83.3	92.6	68.5	63.4	73.7	8.2
√	√	√			88.6	83.8	93.4	69.8	64.6	74.9	10.3
√	√	√	√		90.1	85.5	94.7	69.9	64.6	75.1	9.4
√	√	√	√	√	90.2	85.2	95.3	70.6	65.3	75.9	9.4

**Table 5:** Comparison table of experimental data of each model.

Comparison Model	P/(%)	R/(%)	mAP@0.5/(%)	FLOPs/(G)	Inference Time/(ms)
TOOD	88.3	76.8	86.0	123.0	25.3
Faster R-CNN	87.5	77.4	86.3	134.0	72.5
RTDETR-L	86.1	76.6	84.4	32.0	19.5
RTDETR-R18	86.3	77.0	83.0	19.9	6.7
YOLOv3-tiny	81.9	74.5	79.8	19.0	2.2
YOLOv5	84.1	80.9	85.9	7.2	1.6
YOLOv5-P6	86.7	73.5	83.6	7.3	1.7
YOLOv6	84.8	71.9	80.9	11.9	1.4
YOLOv8	88.8	80.1	87.5	8.2	1.7
YOLOv10	84.9	75.6	85.1	8.2	2.2
YOLOv11	88.2	77.9	86.7	6.3	1.6
FCDHI-YOLOv8	89.8	83.0	90.2	9.4	3.3

## Data Availability

The original contributions presented in this study are included in the article. Further in-quiries can be directed to the corresponding author(s).

## References

[1] Yang L., Chen Y.F., Hao Y.P., Li L.C., Li H., Huang Z.H. (2023). Detection Method for Equivalent Ice Thickness of 500-kV Overhead Lines Based on Axial Tension Measurement and Its Application. IEEE Trans. Instrum. Meas..

[2] Hao Y.P., Huang L., Wei J., Liang W., Pan R.J., Yang L. (2023). The Detecting System and Method of Quasi-Distributed Fiber Bragg Grating for Overhead Transmission Line Conductor Ice and Composite Insulator Icing Load. IEEE Trans. Power Del..

[3] Wang Y., Shao Z.F., Lu T., Wu C.Z., Wang J.M. (2023). Remote Sensing Image Super-Resolution via Multiscale Enhancement Network. IEEE Geosci. Remote Sens. Lett..

[4] Shi J.C., Liu W., Shan H.Y., Li E.Z., Li X., Zhang L.P. (2023). Remote Sensing Scene Classification Based on Multibranch Fusion Attention Network. IEEE Geosci. Remote Sens. Lett..

[5] Ou K.T., Dong C.J., Liu X.K., Zhai Y.K., Li Y., Huang W.X. (2024). Drone-TOOD: A Lightweight Task-Aligned Object Detection Algorithm for Vehicle Detection in UAV Images. IEEE Access.

[6] Khan M.W., Obaidat M.S., Mahmood K., Batool D., Badar H.M.S., Aamir M. (2024). Real-Time Road Damage Detection and Infrastructure Evaluation Leveraging Unmanned Aerial Vehicles and Tiny Machine Learning. IEEE Internet Things..

[7] Ma C.X., Xue F.S. (2024). A Review of Vehicle Detection Methods Based on Computer Vision. J. Intell. Connect. Veh..

[8] Chen S.-L., Chen T.-Y., Mao Y.-C., Lin S.-Y., Huang Y.-Y., Chen C.-A. (2023). Detection of Various Dental Conditions on Dental Panoramic Radiography Using Faster R-CNN. IEEE Access.

[9] Zhou C.J., Wu M.Q., Lam S.-K. (2022). Enhanced Multi-Task Learning Architecture for Detecting Pedestrian at Far Distance. IEEE Trans. Intell. Transp Syst..

[10] Cai Q., Pan Y.W., Yao T., Mei T. (2022). 3D Cascade RCNN: High Quality Object Detection in Point Clouds. IEEE Trans. Image Process..

[11] Zhao L., Wang J.L., Chen Y.H., Yin Q., Rong G.Y., Zhou S.D. (2024). IST-DETR: Improved DETR for Infrared Small Target Detection. IEEE Access.

[12] Liu C.L., Peng S., Liu S.N., Li J.F. (2025). RT-DETR-LGP: An Effective Defect Detection Method for Light Guide Plates via Multiscale Feature Fusion and Knowledge Distillation. IEEE Trans. Instrum. Meas..

[13] Liang C., Wang Z.-Z., Liu X.-L., Zhang P., Tian Z.-W., Qian R.-L. (2024). SDD-Net: A Steel Surface Defect Detection Method Based on Contextual Enhancement and Multiscale Feature Fusion. IEEE Access.

[14] Huang X.H., Zhu J.H., Huo Y. (2024). SSA-YOLO: An Improved YOLO for Hot-Rolled Strip Steel Surface Defect Detection. IEEE Trans. Instrum. Meas..

[15] Zhou W.T., Cai C.T., Li C.M., Xu H., Shi H.C. (2024). AD-YOLO: A Real-Time YOLO Network with Swin Transformer and Attention Mechanism for Airport Scene Detection. IEEE Trans. Instrum. Meas..

[16] Chien C.-T., Ju R.-Y., Chou K.-Y., Xieerke E., Chiang J.-S. (2025). YOLOv8-AM: YOLOv8 Based on Effective Attention Mechanisms for Pediatric Wrist Fracture Detection. IEEE Access.

[17] Liang J., Cheng J. (2025). Mirror Target YOLO: An Improved YOLOv8 Method with Indirect Vision for Heritage Buildings Fire Detection. IEEE Access.

[18] Wang H., Liu C.Y., Cai Y.F., Chen L., Li Y.C. (2024). YOLOv8-QSD: An Improved Small Object Detection Algorithm for Autonomous Vehicles Based on YOLOv8. IEEE Trans. Instrum. Meas..

[19] Dong T.C., Wang T.Y., Li X.F., Hong J.Z., Jing M.Q., Wei T. (2025). A Large Ship Detection Method Based on Component Model in SAR Images. IEEE J. Sel. Top. Appl. Earth Obs. Remote Sens..

[20] Jiao J.G., Liu F.L., Wang Z.G., Zou G.P., Peng Y.K. (2025). Detection and segmentation of overhead transmission line icing images via an improved YOLOv8-seg. Electr Eng.

[21] Kong X.H., Guan H.L., Jiang L., Wang Y.Y., Zhang C. (2024). Icing detection ADSS transmission optical fiber cable based on improved YOLOv8 network. SIViP.

[22] Lu Y.K., Zhao Y.B., Ju Y. (2023). Improved Detection of Ice Accretion Status on Transmission Lines Using YOLOv5s. Front. Comput. Intell. Syst..

[23] Li H., Huang Z.H., Huang H., Mao X.Y., Yang Q., Zhang H.R. Transmission Line Ice-Covering Defect Detection Method Based on Improved YOLOv8. Proceedings of the 2024 6th International Conference on Energy Systems and Electrical Power (ICESEP).

[24] Gu Y.H., Guo Y., Xie W., Wu Z., Dong S.B., Xie G.K. (2025). MDSF: A Plug-and-Play Block for Boosting Infrared Small Target Detection in YOLO-Based Networks. IEEE Trans. Geosci. Remote Sens..

[25] Kim H., Park S., Kim H., Ahn J., Lee T.-Y., Ha Y. (2024). YOLOv7F: Enhanced YOLOv7 WITH Guided Feature Fusion. IEEE Access.

[26] Yang J., Qiu P.J., Zhang Y.C., Marcus D.S., Sotiras A. (2024). D-Net: Dynamic Large Kernel with Dynamic Feature Fusion for Volumetric Medical Image Segmentation. arXiv.

[27] Lang X.M., Han F.C. (2022). MFL Image Recognition Method of Pipeline Corrosion Defects Based on Multilayer Feature Fusion Multiscale GhostNet. IEEE Trans. Instrum. Meas..

[28] Zhao W.X., Syafrudin M., Fitriyani N.L. (2023). CRAS-YOLO: A Novel Multi-Category Vessel Detection and Classification Model Based on YOLOv5s Algorithm. IEEE Access.

[29] Di B., Xiang L., Daoqing Y., Kaimin P. (2024). MARA-YOLO: An Efficient Method for Multiclass Personal Protective Equipment Detection. IEEE Access.

[30] Liu S.T., Huang D., Wang Y.H. (2019). Learning Spatial Fusion for Single-Shot Object Detection. arXiv.

[31] Li K., Zhong X., Han Y. (2025). A High-Performance Small Target Defect Detection Method for PCB Boards Based on a Novel YOLO-DFA Algorithm. IEEE Trans. Instrum. Meas..

[32] Zhang Q., Zhang J.N., Li Y., Zhu G.F., Wang G.F. (2025). ID-YOLO: A Multimodule Optimized Algorithm for Insulator Defect Detection in Power Transmission Lines. IEEE Trans. Instrum. Meas..

[33] Yi W.G., Yang J.W., Yan L.W. (2024). Research on Underwater Small Target Detection Technology Based on Single-Stage USSTD-YOLOv8n. IEEE Access.

[34] Zhang H., Xu C., Zhang S.J. (2023). Inner-IoU: More Effective Intersection over Union Loss with Auxiliary Bounding Box. arXiv.

[35] Zhu X.J., Luan Y.S., Zhao P.H., Tang T., Wu Z.D. (2025). Physical IoU-Based YOLO Network for Shortwave Communication Burst Signal Recognition. IEEE Commun. Lett..

[36] Huang P.P., Tian S.H., Su Y., Tan W.X., Dong Y.F., Xu W. (2024). IA-CIOU: An Improved IOU Bounding Box Loss Function for SAR Ship Target Detection Methods. IEEE J-STARS.

[37] Zheng J.H., Guo J., Hao M. SAR Aircraft Target Detection Based on Improved YOLOv8. Proceedings of the 2024 7th International Con-ference on Pattern Recognition and Artificial Intelligence (PRAI).

[38] Dou J.X., Tan W.X., Huang P.P., Su Y., He Y.X., Zhang J.X., Chen Y.M., Shen Z.K., Li Z. Improved YOLOv8n for Concealed Object Detection in Human Body Millimeter Wave Images. Proceedings of the 2024 IEEE International Conference on Signal, Information and Data Processing (ICSIDP).

[39] The Transmission Line Icing Detection Dataset. https://download.csdn.net/download/kelp123456/90617358.

[40] Kaleem Z. (2025). Lightweight and Computationally Efficient YOLO for Rogue UAV Detection in Complex Backgrounds. IEEE Trans. Aerosp. Electron. Syst..

[41] Fu Z.J., Zhang F., Ren X.Y., Hao B., Zhang X.Y., Yin C.L. (2024). LE-YOLO: Lightweight and Efficient Detection Model for Wind Turbine Blade Defects Based on Improved YOLO. IEEE Access.

[42] Wang Y.R., Song X.K., Feng L.L., Zhai Y.J., Zhao Z.B., Zhang S.Y., Wang Q.M. (2024). MCI-GLA Plug-In Suitable for YOLO Series Models for Transmission Line Insulator Defect Detection. IEEE Trans. Instrum. Meas..

